# Colitis nucleomigrans: The third type of microscopic colitis (part 1)

**DOI:** 10.1111/pin.12996

**Published:** 2020-08-06

**Authors:** Mitsuhiro Tachibana, Tomohiko Hanaoka, Shinya Watanabe, Masahiro Matsushita, Tadahiro Isono, Yutaka Tsutsumi

**Affiliations:** ^1^ Department of Diagnostic Pathology Shimada Municipal Hospital Shizuoka Japan; ^2^ Department of Gastroenterology Shimada Municipal Hospital Shizuoka Japan; ^3^ Department of Surgery Shimada Municipal Hospital Shizuoka Japan; ^4^ Diagnostic Pathology Clinic Pathos Tsutsumi Aichi Japan

**Keywords:** apoptosis, colitis nucleomigrans, diarrhea, immunohistochemistry, microscopic colitis

## Abstract

Microscopic colitis (MC), encompassing collagenous colitis and lymphocytic colitis, is featured by chronic diarrhea, normal‐looking endoscopic findings and unique microscopic appearance. After reviewing biopsied nonspecific colitis, we propose the third type of MC: colitis nucleomigrans (CN). Histopathological criteria of CN included: (i) chained nuclear migration to the middle part of the surface‐lining columnar epithelium; (ii) apoptotic nuclear debris scattered below the nuclei; and (iii) mild/moderate chronic inflammation in the lamina propria. Thirty‐three patients (M:F = 20:13; median age 63 years, range 17–88) fulfilled our criteria. Seven cases demonstrated MC‐like clinical/endoscopic features. Mucosal reddening with or without erosion/aphtha was endoscopically observed in the remaining 26 cases with inflammatory bowel disease (IBD)‐like features: occult/gross hematochezia seen in 19, abdominal pain in two and mucin secretion in two. Cleaved caspase‐3‐immunoreactive apoptotic debris appeared more frequently in IBD‐like CN than in MC‐like CN, while CD8‐positive intraepithelial lymphocytes comparably appeared in both. Proton pump inhibitors (PPIs) were administered in five (71%) cases with MC‐like features, and in three diarrhea improved after drug cessation. In IBD‐like CN cases, eight (31%) received PPIs. Four patients received chemotherapy against malignancies. Four patients associated immune‐related disorders. Microscopic appearance of CN also appeared in a remission state of ulcerative colitis (12/20 lesions).

**Abbreviations**
CN: colitis nucleomigrans
CC: collagenous colitis
IBD: inflammatory bowel disease
LC: lymphocytic colitis
MC: microscopic colitis
NSAIDs: non‐steroid anti‐inflammatory drugs
PPIs: proton pump inhibitors

## INTRODUCTION

Chronic colitis encompasses a variety of categories such as inflammatory bowel disease (IBD), treatment‐related colitis (pseudomembranous colitis, chemical colitis, chemotherapy‐induced colitis and radiation colitis), infectious colitis, ischemic colitis and microscopic colitis (MC).^1^ The appropriate histologic diagnosis of colitis is requested in routine pathology services, and pathologists should thus be cautious about the implication of biopsy diagnosis.

Microscopic colitis predominantly affects middle‐aged women and is featured by chronic non‐bloody watery diarrhea and normal endoscopic appearance of the colorectal mucosa, but with altered microscopic appearance.^2^, ^3^ Other common symptoms include nocturnal diarrhea, abdominal pain and weight loss.^2^, ^3^ The category of MC encompasses two distinct entities such as collagenous colitis (CC) and lymphocytic colitis (LC). They share many clinical features, including the manifestation of clinical symptoms, the association with autoimmune disorders and the response to treatment.^3^, ^4^, ^5^ They differ histologically: CC is featured by deposition of subepithelial collagen fibers ‘collagen bands’ in the uppermost part of the lamina propria mucosae, while a marked increase of CD8‐positive intraepithelial lymphocytes (IELs) is characteristic of LC. Overlapped lesions between CC and LC have been reported.^3^, ^4^, ^5^

In routine pathology practice, we not rarely encounter biopsy specimens displaying microscopic features of chronic nonspecific colitis without collagen bands or increased IELs. In the present study, we propose the third type of MC: colitis nucleomigrans (CN). Histologically, the nuclei of the surface‐lining colorectal columnar cells are migrated in chain to the middle part of the cells, and apoptotic nuclear debris is scattered beneath the chained nuclei. A total of 33 cases were enrolled in the present analysis. Patients manifested chronic non‐bloody watery diarrhea (MC‐like symptoms) with normal endoscopic appearance (n = 7) or microscopic/gross hematochezia, abdominal pain or mucin secretion (IBD‐like symptoms) endoscopically showing mucosal reddening with or without focal erosions (n = 26).

The etiology and pathogenesis of CN is discussed in the present article (part 1), and our paired article (part 2) describes electron microscopic features of CN.^6^

## MATERIALS AND METHODS

### Patients

We retrospectively reviewed colorectal biopsy specimens diagnosed as nonspecific colitis at Shimada Municipal Hospital, Shimada, Shizuoka, Japan, in the period from January 2016 to December 2019. The total number of colorectal biopsy specimens in the four‐year period summed up to 1287. Eligible patents were chosen through a search of pathology records. Hospital charts were then reviewed for extracting patient's age and gender, results of microbial culture of the feces, presenting clinical symptoms, the association with malignancies or autoimmune disorders, the regular use of proton pump inhibitors (PPIs) and other drugs, and the location of biopsies chosen for diagnosis (the cecum through rectum).

The specimens diagnosed as CC were also evaluated in the same period (n = 5, M:F = 5:0; median age 73 years, range 65–82). No case of LC was encountered. The biopsied colorectal mucosa in a remission state of ulcerative colitis (UC) was also studied as reference cases (n = 20; M:F = 9:11; median age 51 years, range 24–79). As a negative control, normal portions of the colorectal mucosa in five surgical specimens of colorectal cancer were evaluated (M:F = 3:2; median age 82 years, range 74–88).

### Diagnosis

The number and location of biopsies were at the discretion of three endoscopists (TH, SW and MM). No endoscopic evidence of active inflammation or neoplastic lesions was noted. Instead, normal endoscopic appearance or patchy mucosal reddening with or without erosion was recorded. Microbial culture of the feces was performed in 10 cases, and nonpathogenic (indifferent) bacteria were identified in four. All biopsies were reviewed by two pathologists (MT and YT), who made a diagnosis of CN based upon the histologic criteria as follows: (i) nuclear migration in chain to the middle part of the surface‐lining columnar epithelium (abundant eosinophilic cytoplasm seen below the nuclei); (ii) fragmented apoptotic figures (nuclear debris) scattered below the nuclei; and (iii) mild to moderate nonspecific chronic inflammation in the lamina propria mucosae. There were no subepithelial collagen bands with the thickness more than 10 μm. IELs were not significantly increased (much less than 20 lymphocytes per 100 surface epithelial cells).

### Immunohistochemical analysis

Apoptotic nuclei were visualized as diaminobenzidine brown deposits with immunoperoxidase staining for cleaved caspase‐3 by using a 1:500‐diluted rabbit polyclonal antibody available from Cell Signaling Technology (Danvers, MA, USA), as was described earlier.^7^ In order to identify IELs, immunostaining for CD3 and CD8 was performed using 1:100‐diluted mouse monoclonal antibodies (clones: LN10 and C8/144B) available from Agilent Technologies (Santa Clara, CA, USA). In order to identify macrophages in the lamina propria, immunostaining for CD68 was performed using a prediluted mouse monoclonal antibody (clone: PG‐M1) available from Nichirei Biosciences (Tokyo, Japan). The nuclei were briefly counterstained with Mayer's hematoxylin. Diaminobenzidine‐labelled apoptotic bodies and IELs among surface‐lining epithelial cells were quantified as follows. The numbers of cleaved caspase‐3‐positive apoptotic bodies and CD8‐immunoreactive IELs were counted among at least 170 colorectal surface‐lining epithelial cells in the most severely involved areas, and the results were expressed the number per 100 epithelial cells. A cluster of cleaved caspase 3‐positive nuclear debris seemingly derived from a single cell was calculated as one.

### Ethics approval

All the procedures were in accordance with the ethical standards of the responsible institutional committee on human experimentation and with the Helsinki Declaration of 1964 and later versions. Written informed consent was obtained from the respective patients after selection of cases, including negative control cases. The study was approved in December 2019 by the Ethics Committee for Clinical Research of Shimada Municipal Hospital, Shimada, Shizuoka, Japan (approval number R01‐10).

## RESULTS

### Clinical and endoscopic features of CN

**Figure 1:**
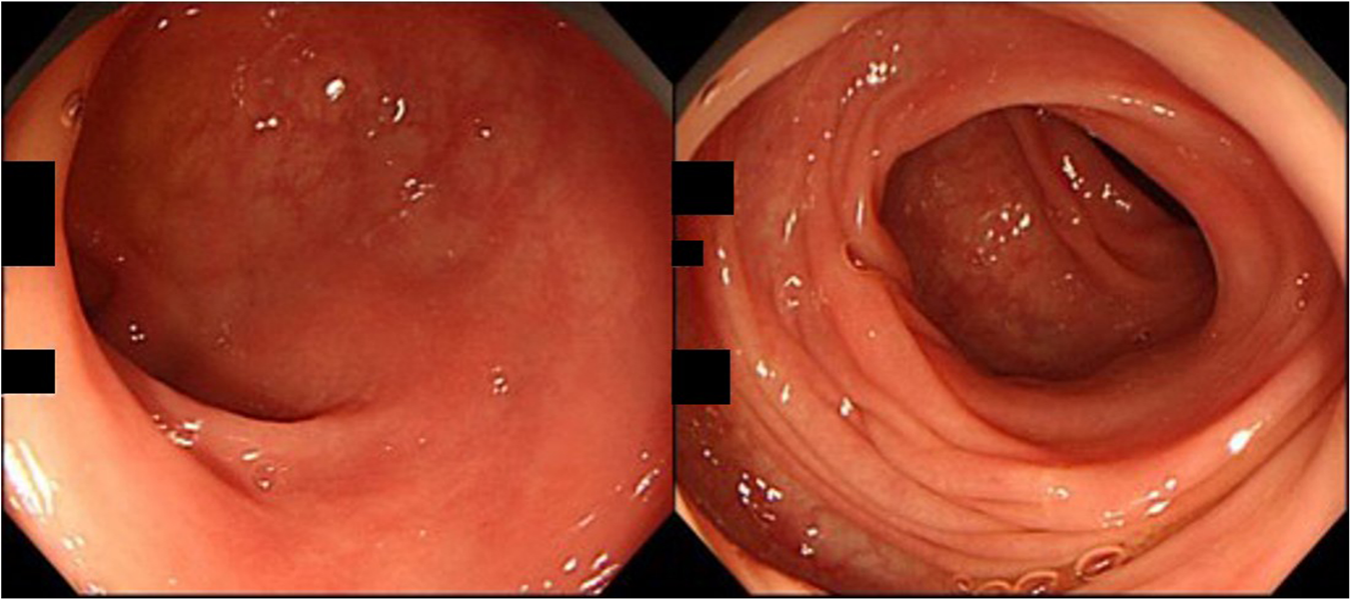
Colonoscopic findings in a patient of colitis nucleomigrans (CN) with microscopic colitis (MC)‐like symptoms (67 year‐old female; left, rectum; right, sigmoid colon). The colorectal mucosa appears normal without mucosal edema, reddening, erosion or ulceration.

**Figure 2:**
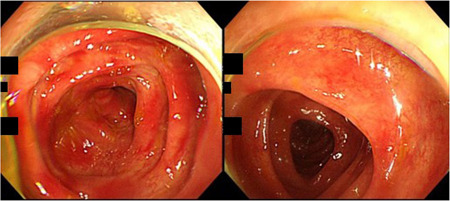
Colonoscopic findings in a patient of colitis nucleomigrans (CN) with inflammatory bowel disease (IBD)‐like symptoms (54 year‐old male; left, ascending colon; right, transverse colon). The colonic mucosa displays multiple patchy reddening.

Thirty‐three patients fulfilled our criteria of CN as described above. The frequency of CN among colorectal biopsy specimens was 33/1287 (2.6%). Twenty males and 13 females were enrolled, and the age ranged from 17 to 88 years with the mean 58.4 and the median 63. Patients manifested chronic non‐bloody watery diarrhea (MC‐like symptoms) with normal endoscopic appearance (n = 7; Fig. 1) or occult (n = 9)/gross (n = 10) hematochezia, abdominal pain (n = 2) or mucin secretion (n = 2) (IBD‐like symptoms) endoscopically showing mucosal reddening with or without focal erosion/aphtha (n = 26; Fig. 2). One patient in the MC‐like group showed fecal occult blood, while normal‐looking endoscopic findings were recorded in three patients in the IBD‐like group.

Proton pump inhibitors were regularly administered in 13 patients; five (71%) with MC‐like features and eight (31%) with IBD‐like features. In the MC‐like group, lansoprazole was administered in two and others (esomeprazole and rabeprazole) in three. In three patients whose diarrhea was improved after cessation of the drug, non‐lansoprazole medication was given. In the IBD‐like group, lansoprazole was used in two, esomeprazole in two, rabeprazole in three and vonoprazan in one.

Histamine H_2_‐blockers and non‐steroidal anti‐inflammatory drugs (NSAIDs) were used in two patients each in the IBD‐like group. Hypertension was recorded in eight patients. Two patients were suspected of Behçet disease, but without intestinal ulceration. One patient suffered from IgA nephropathy, and one patient associated Sjögren syndrome. Four patients received chemotherapy against plasma cell myeloma, nodal diffuse large B‐cell lymphoma, splenic diffuse large B‐cell lymphoma, or surgically resected colonic mucinous adenocarcinoma. The periods between the completion of chemotherapy and the diagnosis of CN were 0, 5, 6 and 53 months, respectively.

Clinical and endoscopic features of CN with MC‐like features (n = 7) and CN with IBD‐like features (n = 26) are summarized in Table 1.

**Table 1:** Summary of cases of colitis nucleomigrans

Age (yrs) /gender	Locations	Watery diarrhea	IBD‐like features	Endoscopic findings	C‐caspase 3 (/100 epithelial cells)	CD8+IELs (/100 epithelial cells)	Medication	Microbial culture	Concomitant disease
(i) Cases with microscopic colitis‐like features									
61/F	A,T,D	3+	FOB	normal	2.3	17.4	Esomeprazole^†^		
26/M	A	3+	None	Normal	22.6	13.9		Negative	
32/F	C,A,D,S,R	3+	None	Normal	4.3	5.0	Lansoprazole	*Pseudomonas aeruginosa*	
65/M	S,R	3+	None	Normal	20.6	3.4	Rabeprazole^†^	*Bacillus subtilis*	
67/F	A,T,D,S,R	3+	None	Normal	11.2	2.3	Esomeprazole^†^		Splenic DLBCL
75/M	S	3+	None	Normal	9.9	3.5		Negative	HT
80/M	A,T	3+	None	Mucosal edema	1.4	3.6	Lansoprazole	Negative	
(ii) Cases with inflammatory bowel disease‐like features									
36/F	T,D	0	FOB	Normal	19.4	7.4			Behçet disease
58/M	D,S,R	0	FOB	Normal	40.4	14.1		NTM	
46/F	S,R	0	FOB	Reddening	36.6	1.7		Negative	HT
59/M	S,R	2+	FOB	Reddening	35.8	6.7	Rabeprazole		Plasma cell myeloma
70/M	T	0	FOB	Reddening	7.0	4.2			
79/M	R	0	FOB	Reddening	37.4	1.6	Rabeprazole		HT
55/M	R	0	FOB	Erosion	28.4	17.2	Esomeprazole		
46/M	S,R	0	FOB	FIV & MR	8.0	4.6	H_2_ blocker		HT, IgA nephropathy
88/M	C	0	FOB	Ischemia ‐like change	27.3	15.6	NSAIDs		
17/M	S,R	0	Melena	Normal	5.8	9.3			
17/M	A	0	Melena	Reddening	52.8	5.6	Lansoprazole	Negative	
30/F	S	0	Melena	Reddening	7.3	5.4			
47/M	T	0	Melena	Reddening	64.8	9.1			
54/M	T,D	0	Melena	Reddening	7.9	11.7	H_2_ blocker		
65/F	D	0	Melena	Reddening	25.7	7.5	Rabeprazole		
66/F	S	0	Melena	Reddening	16.5	4.9			
76/F	S	0	Melena	Reddening	48.6	5.2			HT
85/M	S	0	Melena	Reddening	5.6	4.0	Esomeprazole, NSAIDs		Sjögren syndrome
76/M	A	0	Melena	Erosion	21.5	8.5			HT
50/F	S	0	Abdominal pain	Reddening	34.6	3.1			
63/F	S	0	Abdominal pain & mucin secretion	Reddening	4.9	10.5		*E. coli* (O‐15)	
70/F	D,R	0	Mucin secretion	Reddening	7.7	9.6			
46/M	S,R	1+	Weight loss	Reddening	36.6	1.7	Lansoprazole		HT
82/M	C,A,T,D,S	3+	Weight loss	Ischemia ‐like change	1.8	9.3			HT
64/F	S	0	Detailed check for colon cancer	Erosion	19.0	8.2	Vonoprazan		Colon cancer
77/M	S,R	0	Detailed check for Behçet disease	Erosion & aphtha	29.2	10.7		Negative	HT, Behçet disease, nodal DLBCL

Abbreviations: 1+, mild; 2+, moderate; 3+, severe; A, ascending colon; C, cecum; C‐caspase 3, cleaved caspase‐3; D, descending colon; DLBCL, diffuse large B‐cell lymphoma; F, female; FOB, fecal occult blood; FIV, focal indistinct vascular pattern; GIST, gastrointestinal stromal tumor; HT, hypertension; IBD, inflammatory bowel disease; IELs, intraepithelial lymphocytes; M, male; MR, mucosal roughening; NTB, non‐tuberculous mycobacteria; NSAIDs, non‐steroidal anti‐inflammatory drugs; R, rectum; S, sigmoid colon; T, transverse colon; watery diarrhea score: 0, negative; yrs, years.

† Diarrhea improved after cessation of proton pump inhibitors.

### Histopathological features

The microscopic criteria of CN were solely dependent upon the hematoxylin and eosin histology. Chained nuclear migration to the middle part of the columnar cells was quite characteristic and easily recognizable microscopically. Nuclear migration was confined to the surface‐lining columnar cells, and never involved the crypt epithelial cells. As a result of chained nuclear migration in the surface‐lining columnar cells, eosinophilic cytoplasm was evident beneath the nuclei as an eosinophilic band‐like zone. Apoptotic nuclear debris was scattered in the eosinophilic zone of the cytoplasm. Intraepithelial lymphocytes were also distributed in the same zone. The degree of chronic inflammation in the lamina propria mucosae was mild to moderate. Both small lymphocytes and plasma cells consist of the main mononuclear cell components. Eosinophils were scarcely seen.

**Figure 3:**
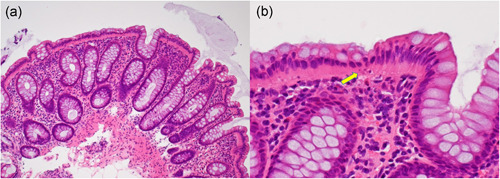
Microscopic findings of colitis nucleomigrans (CN) with inflammatory bowel disease (IBD)‐like symptoms (70 year‐old male; HE). (**a**) The nuclei of the surface‐lining columnar cells are migrated in chain to the middle part of the cells. Eosinophilic cytoplasm is evident beneath the chained nuclei. The colonic crypts are arranged in parallel without features of cryptitis or nuclear migration. The lamina propria mucosae reveals moderate chronic inflammatory infiltration. (**b**) High‐powered view reveals the migrated chained nuclei in the surface‐lining columnar epithelial cells. Beneath the nuclei, apoptotic bodies (clustered fragments of nuclear debris) are observed (arrow). Neither subepithelial collagen bands nor increased intraepithelial lymphocytes (IELs) are recognized.

The total number of biopsy pieces with microscopic features of CN sampled from 33 cases summed up to 65. The microscopic features of CN were seen in any part of the large‐intestine (cecum 3, ascending colon 7, transverse colon 10, descending colon 8, sigmoid colon 21 and rectum 16). The chained nuclear migration was never observed in the crypt epithelial cells. Neither collagen bands nor increased IELs (>20 IELs among 100 epithelial cells) were noted in the specimens. Representative microscopic appearance of CN is demonstrated in Fig. 3.

In the same period of study, we experienced five cases of CC but no case of LC. The colorectal mucosa in a remission state of UC (n = 20), as well as normal colorectal mucosa sampled from surgical specimens of colorectal cancer (n = 5), were also evaluated. The CN‐type microscopic features were not seen in CC and control normal colorectal mucosa, but observed in twelve (60%) lesions in UC in a remission state.

### Immunohistochemical findings

Immunohistochemically, IELs were immunoreactive for both CD3 and CD8. IELs were scattered among the CN lesion (but not significantly increased). The average numbers of CD8‐positive IELs were 7.0 per 100 surface epithelial cells (median 3.6, range 2.3–17.4) in CN with MC‐like features, and 7.9 (median 7.9, range 1.6–17.2) in CN with IBD‐like features. In the control groups, the average numbers of IELs were 18.1 (median 7.2, range 4.0–64.9) in CC, 7.6 (median 6.0, range 0–18.3) in UC in a remission state, and 4.4 (median 4.2, range 1.3–7.1) in surgically resected normal colorectal mucosa.

**Figure 4:**
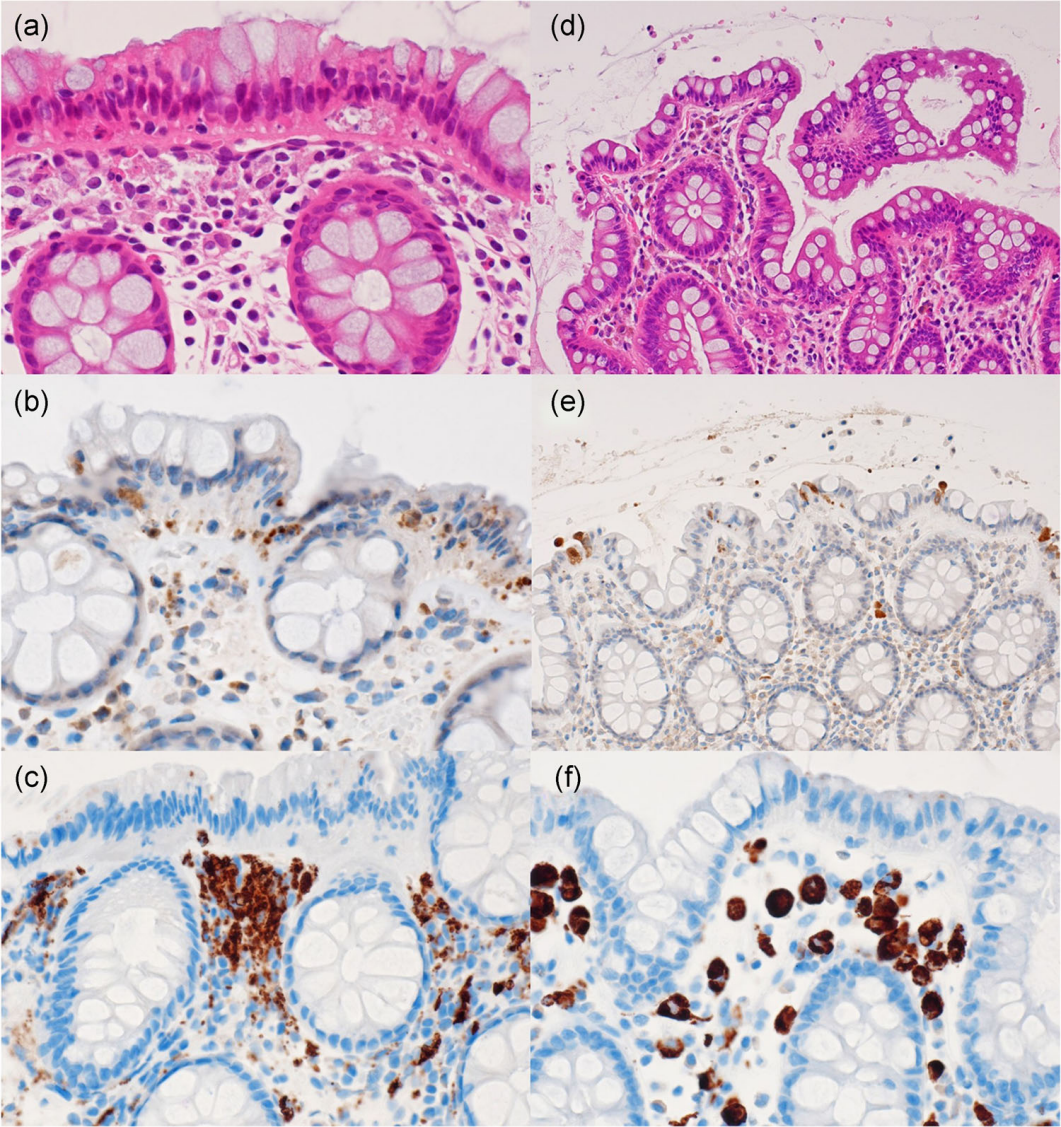
Comparative immunohistochemical features of colitis nucleomigrans (CN) with microscopic colitis (MC)‐like features (**a**‐**c**, 67‐year‐old female) and control normal colon (**d**‐**f**, 74‐year‐old male): HE (**a, d**), cleaved caspase‐3 (**b, e**) and CD68 (**c, f**). In CN, fragmented apoptotic bodies, clustered beneath the chained migrated nuclei, are immunoreactive for cleaved caspase‐3. In contrast, in the surgically removed normal colorectal mucosa, cleaved caspase‐3‐immunoreactive rounded nuclei are extruded onto the lumen. CD68‐positive macrophages located in the upper part of the lamina propria mucosae actively phagocytize apoptotic debris in the plump and amebic cytoplasm, while macrophages in the normal mucosa are round in shape and smaller in size.

Apoptotic bodies with a ‘debris pattern’ (clustered fragments of nuclear debris) beneath the chained migrated nuclei in CN were immunoreactive for cleaved caspase‐3 (Fig. 4a, b). In the MC‐like CN lesions, cleaved caspase‐3‐positive apoptotic bodies of debris type counted 10.3 per 100 surface‐lining epithelial cells on average (median 9.9, range 1.4–22.6), while the average number in the IBD‐like CN lesions was higher: 28.2 (median 20.5, range 1.8–64.8).

In contrast, in surgically removed normal colorectal mucosa, cleaved caspase‐3‐immunoreactivity was observed mainly as non‐fragmented apoptotic nuclei along the luminal space on the surface‐lining cells, as an ‘extrusion pattern’ (Fig. 4c, d). Apoptosis of debris type was infrequently noted in normal colorectal mucosa. Apoptosis of extrusion type was occasionally observed also in the CN lesions. The number of cleaved caspase 3‐immunoreactive apoptotic bodies of extrusion type in the normal colorectal mucosa ranged from 1.0 to 5.4 (mean 3.4, median 3.5) per 100 epithelial cells. The apoptotic bodies in CC (n = 5) predominantly belonged to debris type and the number ranged from 6.1 to 23.4 (mean 14.3, median 13.5). In the lesions in UC in a remission state, epithelial apoptosis was identified in a mixed debris and extrusion pattern: The number of apoptosis of debris type ranged from 6.2 to 63.2 (mean 29.1, median 28.2).

The CD68‐positive macrophages located in the upper part of the lamina propria mucosae occasionally phagocytized cleaved caspase 3‐immunoreactive apoptotic bodies (fragmented nuclear debris) in the cytoplasm. Such a pattern was seen in any type of the colorectal lesions and in normal colorectal mucosa, but their number was increased in the lesion of CN (Fig. 4e, f). The CD68‐positive macrophages in CN showed the plump and amebic cytoplasm, while those in the normal colorectal mucosa tended to be round in shape and smaller in size. The number of apoptotic debris‐phagocytizing CD68‐positive macrophages in CN with IBD‐like features was comparable with that in CN with MC‐like features.

## DISCUSSION

Recent epidemiological studies suggest that MC is more common than initially expected: one study identified MC in 10% of colonic biopsy specimens from patients with non‐bloody diarrhea, and in >20% of such patients older than 70 years.^3^, ^5^ Reportedly, MC occurs more frequently in females than in males and usually affects patients in the sixth and seventh decades.^3^, ^5^, ^8^ In the present analysis, however, all the cases of CC were male. It is of note that in a considerable percentage of MC cases, a range of endoscopic abnormalities was reported, such as patchy reddening/inflammation, alteration of the vascular mucosal pattern, mosaic pattern, diffuse mucosal cloudiness, mucosal nodularity or mucosal defects.^9^, ^10^, ^11^, ^12^ The etiology of MC remains unsettled. Oral medications have been suggested as potential triggers: the drugs include lansoprazole, simvastatin, flutamide, ranitidine, carbanazepin, vinburninem paroxetine, sertraline, penicillin V, pivmecillinam, orlistate, tardyferon and Cyclo 3 Fort.^13^ In particular, intake of lansoprazole, a representative drug in PPIs, is closely associated with collagenous colitis.^9^, ^14^

We herein proposed the third type MC histologically characterized by chained migrated nuclei in the colorectal surface‐lining epithelium, in association with accelerated apoptosis of debris type beneath the nuclei. We dared to name this‐type of lesion as colitis nucleomigrans (CN), *nucleus* + *migro* in Latin. Patients with CN were categorized into two clinical/endoscopic subtypes: one group manifested MC‐like symptoms (watery diarrhea) with minimal endoscopic abnormality, while another group is featured by IBD‐like symptoms (occult/gross hematochezia, abdominal pain or mucin secretion) with patchy reddening of the colorectal mucosa with or without erosion/aphtha.

When compared with conventional MC (namely, CC and LC), CN showed a male predominance, with a wide age range from 17 to 88 years, with the median age of 63 years. Some patients received medication of PPIs as a potential trigger, as is so in conventional MC. PPIs were administered in eight of 26 cases of CN with IBD‐like features: lansoprazole in two, esomeprazole in two, rabeprazole in three and vonoprazan in one. In five of seven cases of CN with MC‐like features, lansoprazole was administered in two and others (esomeprazole and rabeprazole) in three. Interestingly enough, in all the three patients whose diarrhea was improved after cessation of the drug, non‐lansoprazole medication was given. Further clinicopathological studies are definitely requested to clarify the causal relationship between MC‐like CN and non‐lansoprazole treatment. Pharmacokinetic and pharmacodynamic studies have described that the metabolic pathway is different between lansoprazole and other types of PPIs.^15^, ^16^

In four patients (one in MC‐like type, and three in IBD‐like type), CN occurred during or several months after chemotherapy against hematopoietic malignancies or surgically resected colonic adenocarcinoma. Regarding autoimmune disorders, two patients of CN were clinically suspected of Behçet disease without intestinal ulceration, and two patients suffered from IgA nephropathy and Sjögren syndrome, respectively. In fact, MC is often associated with autoimmune disorders in a range of 20–60% for both CC and LC.^2^, ^3^, ^4^, ^13^ The candidate etiological factors of CN should thus include the medication of PPIs or anti‐cancer drugs and the association of immune‐related disorders.

We suppose that abnormality of epithelial apoptosis in the colorectal mucosa may be linked to the pathogenesis of CN. In the intestinal mucosa, the microbiotas may provoke apoptosis of epithelial cells in balance with adaptive immune homeostasis. Nakahashi‐Oda *et al*.^17^ recently documented that apoptotic epithelial cells negatively regulate the gut commensal bacteria‐stimulated proliferation of regulatory T‐cells, playing a central role in the maintenance of gut tissue homeostasis: namely, apoptotic epithelial cells suppress the proliferation of regulatory T‐cells to regulate mucosal homeostasis. It is also known that gut commensal bacteria play an important role in autoimmune disorders.^18^ In fact, dysbiosis of the gut normal flora has been observed in patients with autoimmune disorders, such as systemic lupus erythematosus.^18^ On the other hand, some microbiotas, especially *Clostridium* IV or XIV, may induce regulatory T‐cells and inhibit the development of IBD.^19^

The chained nuclear migration to the middle part of the surface‐lining epithelial cells was quite unique and pathognomonic of CN, and easily recognizable in hematoxylin and eosin‐stained biopsy specimens. Such microscopic appearance of CN was scarcely observed in CC and the control normal colorectal mucosa. To the best of our knowledge, the chained nuclear migration in the columnar epithelial cells is scarcely seen in other epithelial tissue, we believe. The mechanisms of nuclear migration remain unknown. It is possible that abnormality of cytoskeletal proteins may impair localization of the nuclei that are normally anchored to the basal part of the columnar cells. However, negative electron microscopic findings are presented in our paired article (part 2).^6^

It is of note that under the low‐powered magnification, pathologists may confuse CN with CC, because the eosinophilic cytoplasmic component beneath the migrated nuclei of the surface epithelial cells looks like the collagen bands in CC at a glance. Therefore, close microscopic observation under a high‐powered magnification is requested to avoid inappropriate diagnosis.

More than half (60%) of colorectal biopsy specimens sampled from patients with UC in a remission state showed the microscopic appearance resembling CN. We consider that chained nuclear migration to the middle part of the surface‐lining columnar cells may be correlated with abnormality in apoptotic processes of the colorectal epithelial cells. Further clinicopathological studies are needed to evaluate whether or not CN can really be regarded as a distinct disease entity in the category of MC.

The mode of apoptosis in normal gut mucosa has been analyzed microscopically. Sträter *et al*.^20^ described two different patterns of enterocytic apoptosis: (i) apoptotic bodies were engulfed by adjacent epithelial cells; and (ii) apoptotic cells with only subtle morphological changes were extruded into the gut lumen. The engulfment pattern was seen predominantly in the crypt. The extrusion pattern was restricted to the luminal mucosal surface. Iwanaga *et al*.^21^ reported unique features of apoptosis at the tip of small intestinal villi of the guinea pig. The apical cytoplasmic plates without containing nuclei were pinched off into the lumen, and the nuclei were engulfed by macrophages located in the upper part of the lamina propria mucosae. The fate of apoptotic nuclei of enterocytes may thus be dependent on the site in the mucosa and animal species. In the present study, the protrusion pattern of apoptosis was seen in the normal colorectal mucosa, just in accordance with the report by Sträter *et al*.^20^ In CN, CD68‐positive macrophages possessed the plump and amebic cytoplasm and actively phagocytized apoptotic nuclear debris, while those in the normal colorectal mucosa tended to be round in shape and smaller in size. Abnormality of enterocytic apoptosis has so far been linked to a variety of intestinal disorders.^22^, ^23^ The chained nuclear migration in the surface‐lining columnar cells, in association with fragmented nuclear debris beneath the nuclei, may represent an accelerated and altered apoptosis (switching from the extrusion type to debris type) in the diseased colorectal mucosa of CN.

Intraepithelial lymphocytes of CD8‐positive cytotoxic T‐cell type are known to promote epithelial apoptosis by secretion of cytotoxic molecules such as granzymes and perforin.^24^ Granzyme B accelerates apoptosis through both caspase‐dependent and ‐independent pathways.^24^ IELs also contribute to cryptal apoptosis and ulceration in active IBD.^25^ In celiac disease, increase of IELs expressing first apoptosis signal (Fas) ligand (CD95L) and perforin provokes mucosal damage and epithelial apoptosis.^26^, ^27^ In acute human immunodeficiency virus infection, both apoptotic epithelial cells and IELs are increased.^28^ In the present study, cleaved caspase‐3‐immunoreactive fragmented apoptotic bodies were more frequently observed in IBD‐like CN than in MC‐like CN, while the number of CB8‐positive IELs and CD68‐positive phagocytizing lamina proprial macrophages was not significantly altered. Supposedly, accelerated apoptosis of debris type may be related to clinical association of occult/gross hematochezia. Lu *et al*.^29^ recently showed in the UC rat model that microRNA‐21‐5p inhibited interleukin‐6 receptor/signal transducer and activator of transcription signal‐mediated pathway to decrease the level of inflammatory cytokines and apoptosis. They thus suggested microRNA‐21‐5p as a candidate of therapeutic molecular target of UC in the human. It is of interest if the microRNA‐21‐5p‐targeted therapy may be applied to CN, especially the IBD‐like subtype.

The clinical and epidemiological features of MC have been established during the past 30 years.^2^, ^3^ In the present analysis, we dared to propose a new variant of MC, ‘CN’. Further clinicopathological studies are needed, in order to settle unanswered questions in this perplexing condition, including the cause, pathophysiology and optimal treatment.

## DISCLOSURE STATEMENT

None declared.

## AUTHOR CONTRIBUTIONS

Each author has participated sufficiently in the work to take public responsibility for appropriate portions of the content: MT and YT designed the study, analyzed histopathological features and drafted the manuscript. YT proposed a basic idea of ‘CN’. TH, SW, MM and TI, attending endoscopists and surgeon of the present case series, earnestly discussed clinical problems. All authors read and approved the final manuscript.

## References

[1] Choi EK , Appelman HD . Chronic colitis in biopsy samples. Surg Pathol Clin. 2017; 10: 841–61. 29103536 10.1016/j.path.2017.07.005

[2] Tysk C , Bohr J , Nyhlin N , Wickbom A , Eriksson S . Diagnosis and management of microscopic colitis. World J Gastroenterol 2008; 14: 7280–88. 19109861 10.3748/wjg.14.7280 PMC2778111

[3] Park T , Cave D , Marshall C . Microscopic colitis: A review of etiology, treatment and refractory disease. World J Gastroenterol 2015; 21: 8804–10. 26269669 10.3748/wjg.v21.i29.8804 PMC4528022

[4] Baert F , Wouters K , D'haens G *et al* Lymphocytic colitis: A distinct clinical entity? A clinicopathological confrontation of lymphocytic and collagenous colitis. Gut 1999; 45: 375–81. 10446105 10.1136/gut.45.3.375 PMC1727642

[5] Williams JJ , Beck PL , Andrews CN , Hogan DB , Storr MA . Microscopic colitis: A common cause of diarrhoea in older adults. Age Ageing 2010; 39: 162–68. 20065357 10.1093/ageing/afp243

[6] Tachibana M , Tsutsumi Y. , Colitis nucleomigrans: The third type of microscopic colitis (part 2). An ultrastructural study. Pathol Int 2020; 70 10.1111/pin.12995 PMC7689711 32761883

[7] Tsutsumi Y , Kamoshida S . Pitfalls and caveats in histochemically demonstrating apoptosis. Acta Histochem Cytochem 2003; 36: 271–80.

[8] Olesen M , Eriksson S , Bohr J , Järnerot G , Tysk C . Microscopic colitis: A common diarrhoeal disease. An epidemiological study in Orebro, Sweden, 1993–1998. Gut 2004; 53: 346–50. 14960513 10.1136/gut.2003.014431 PMC1773978

[9] Chiba M , Sugawara T , Tozawa H *et al* Lansoprazole‐associated collagenous colitis: Diffuse mucosal cloudiness mimicking ulcerative colitis. World J Gastroenterol 2009; 15: 2166–69. 19418592 10.3748/wjg.15.2166 PMC2678590

[10] Cimmino DG , Mella JM , Pereyra L *et al* A colorectal mosaic pattern might be an endoscopic feature of collagenous colitis. J Crohns Colitis 2010; 4: 139–43. 21122497 10.1016/j.crohns.2009.09.004

[11] Koulaouzidis A , Saeed A . Distinct colonoscopy findings of microscopic colitis: Not so microscopic after all? World J Gastroenterol 2011; 17: 4157–65. 22072846 10.3748/wjg.v17.i37.4157 PMC3209563

[12] Moore M , Coleman HG , Allen PB , Loughrey MB . Microscopic colitis: A population‐based case series over a 9‐year period in Northern Ireland. Colorectal Dis 2018; 20: 1020–27. 29742325 10.1111/codi.14247

[13] Chande N , Driman DK , Reynolds RPE . Collagenous colitis and lymphocytic colitis: Patient characteristics and clinical presentation. Scandinav J Gastroenterol 2005; 40: 343–47. 15932175 10.1080/00365520510011623

[14] Thomson R , Thomson RD , Lestina LS *et al* Lansoprazole‐associated microscopic colitis: A case series. Am J Gastroenterol 2002; 97: 2908–13. 12425567 10.1111/j.1572-0241.2002.07066.x

[15] Robinson M , Horn J . Clinical pharmacology of proton pump inhibitors: What the practising physician needs to know. Drugs 2003; 63: 2739–54. 14664653 10.2165/00003495-200363240-00004

[16] Shin JM , Kim N . Pharmacokinetics and pharmacodynamics of the proton pump inhibitors. J Neurogastroenterol Motil 2013; 19: 25–35. 23350044 10.5056/jnm.2013.19.1.25 PMC3548122

[17] Nakahashi‐Oda C , Udayanga KG , Nakamura Y et al. Apoptotic epithelial cells control the abundance of Treg cells at barrier surfaces. Nat Immunol 2016; 17: 441–50. 26855029 10.1038/ni.3345

[18] Bach JF . The hygiene hypothesis in autoimmunity: The role of pathogens and commensals. Nat Rev Immunol 2018; 18: 105–20. 29034905 10.1038/nri.2017.111

[19] Honda K , Littman DR . The microbiota in adaptive immune homeostasis and disease. Nature 2016; 535: 75–84. 27383982 10.1038/nature18848

[20] Sträter J , Koretz K , Günthert AR , Möller P . *In situ* detection of enterocytic apoptosis in normal colonic mucosa and in familial adenomatous polyposis. Gut 1995; 37: 819–25. 8537054 10.1136/gut.37.6.819 PMC1382945

[21] Iwanaga T , Han H , Adachi K , Fujita T . A novel mechanism for disposing of effete epithelial cells in the small intestine of guinea pigs. Gastroenterology 1993; 105: 1089–97. 8405853 10.1016/0016-5085(93)90953-a

[22] Ramachandran A , Madesh M , Balasubramanian KA . Apoptosis in the intestinal epithelium: Its relevance in normal and pathophysiological conditions. J Gastroenterol Hepatol 2000; 15: 109–20. 10735533 10.1046/j.1440-1746.2000.02059.x

[23] Negroni A , Cucchiara S , Stronati L . Apoptosis, necrosis, and necroptosis in the gut and intestinal homeostasis. Mediators Inflamm 2015; 2015: 250762. 26483605 10.1155/2015/250762 PMC4592906

[24] Henkart PA . Lymphocyte‐mediated cytotoxicity: Two pathways and multiple effector molecules. Immunity 1994; 1: 343–46. 7882166 10.1016/1074-7613(94)90063-9

[25] Mitomi H , Ohkura Y , Yokoyama K *et al* Contribution of TIA‐1+ and granzyme B+ cytotoxic T lymphocytes to cryptal apoptosis and ulceration in active inflammatory bowel disease. Pathol Res Pract 2007; 203: 717–23. 17869012 10.1016/j.prp.2007.06.007

[26] Ciccocioppo R , Di Sabatino A , Parroni R *et al* Cytolytic mechanisms of intraepithelial lymphocytes in coeliac disease (CoD). Clin Exp Immunol 2000; 120: 235–40. 10792370 10.1046/j.1365-2249.2000.01200.x PMC1905653

[27] Ciccocioppo R , Di Sabatino A , Parroni R et al. Increased enterocyte apoptosis and Fas‐Fas ligand system in celiac disease. Am J Clin Pathol 2001; 115: 494–503. 11293896 10.1309/UV54-BHP3-A66B-0QUD

[28] Epple HJ , Allers K , Tröger H et al. Acute HIV infection induces mucosal infiltration with CD4+ and CD8+ T cells, epithelial apoptosis, and a mucosal barrier defect. Gastroenterology 2010; 139: 1289–300. 20600014 10.1053/j.gastro.2010.06.065

[29] Lu X , Yu Y , Tan S . The role of the miR‐21‐5p‐mediated inflammatory pathway in ulcerative colitis. Exp Ther Med 2020; 19: 981–89. 32010260 10.3892/etm.2019.8277 PMC6966149

